# Effectiveness of service linkages in primary mental health care: a narrative review part 1

**DOI:** 10.1186/1472-6963-11-72

**Published:** 2011-04-11

**Authors:** Jeffrey D Fuller, David Perkins, Sharon Parker, Louise Holdsworth, Brian Kelly, Russell Roberts, Lee Martinez, Lyn Fragar

**Affiliations:** 1 School of Nursing and Midwifery, Flinders University, Adelaide, Australia; 2 Northern Rivers University Department of Rural Health, School of Public Health, Sydney University, Lismore, Australia; 3 Broken Hill University Department of Rural Health, School of Public Health, Sydney University, Broken Hill, Australia; 4 Research Consultant, Sydney, Australia; 5 School of Tourism & Hospitality Management, Centre for Gambling Education & Research, Southern Cross University, Lismore, Australia; 6 Faculty of Medicine, University of Newcastle, Newcastle, Australia; 7 Greater Western Area Health Service, Orange, New South Wales, Australia; 8 South Australian Department of Health, Adelaide, Australia; 9 Australian Centre for Agricultural Health and Safety, School of Public Health, Sydney University, Moree, Australia

**Keywords:** Narrative review, mental health services, primary health care, cooperative behaviour

## Abstract

**Background:**

With the move to community care and increased involvement of generalist health care providers in mental health, the need for health service partnerships has been emphasised in mental health policy. Within existing health system structures the active strategies that facilitate effective partnership linkages are not clear. The objective of this study was to examine the evidence from peer reviewed literature regarding the effectiveness of service linkages in primary mental health care.

**Methods:**

A narrative and thematic review of English language papers published between 1998 and 2009. Studies of analytic, descriptive and qualitative designs from Australia, New Zealand, UK, Europe, USA and Canada were included. Data were extracted to examine what service linkages have been used in studies of collaboration in primary mental health care. Findings from the randomised trials were tabulated to show the proportion that demonstrated clinical, service delivery and economic benefits.

**Results:**

A review of 119 studies found ten linkage types. Most studies used a combination of linkage types and so the 42 RCTs were grouped into four broad linkage categories for meaningful descriptive analysis of outcomes. Studies that used multiple linkage strategies from the suite of "direct collaborative activities" plus "agreed guidelines" plus "communication systems" showed positive clinical (81%), service (78%) and economic (75%) outcomes. Most evidence of effectiveness came from studies of depression. Long term benefits were attributed to medication concordance and the use of case managers with a professional background who received expert supervision. There were fewer randomised trials related to collaborative care of people with psychosis and there were almost none related to collaboration with the wider human service sectors. Because of the variability of study types we did not exclude on quality or attempt to weight findings according to power or effect size.

**Conclusion:**

There is strong evidence to support collaborative primary mental health care for people with depression when linkages involve "direct collaborative activity", plus "agreed guidelines" and "communication systems".

## Background

The first Australian National Mental Health Policy [1] in 1992 set out to move care from institutions to mainstream health and welfare services. Since that time the importance of partnerships between different health and human service sectors has been promoted. The 1998 Second National Mental Health Plan [2] and the 2004 Australian National Mental Health Strategy [3] called for joint planning, coordination of services and the development of links between different providers. This was further articulated in the Council of Australian Governments (COAG) National Action Plan for Mental Health [4] and most recently in the Fourth National Mental Health Plan[5]. In 2009 the Australian National Health and Hospitals Reform Commission reported that access to and collaboration between support services are key to recovery and self determination for people with mental illness [6]. Australian programs to promote greater primary mental health care involvement in General Practitioner (GP) training and access to allied mental health professionals have been implemented in the past decade [7].

Although mental and physical problems are highly interconnected, western treatment systems tend to be structured in ways that inhibit effective connected care [8]. Hence, even though policies continue to emphasise the importance of effective mental health linkages between primary care (PC), specialist and community health services, the form these linkages should take remains unclear. This narrative review was conducted in response to key national government policy priorities relating to the need for improved service linkages in the Australian health care system. The first objective was to examine evidence from the international literature about the effectiveness of linkages and combinations of linkages in primary mental health care. The second objective was to describe the factors that enable the development of these linkages, which is reported in a companion paper.

## Methods

The study followed the narrative review and thematic synthesis approaches recommended as ways to draw on a range of quantitative and qualitative evidence for the support of decision making by policy makers [9-11]. A review reference group of eight senior policy and service managers in Australian primary mental health care helped guide the review, interpret the findings and assist with the formulation of recommendations.

### Search strategy

A comprehensive search of biomedical, psychological and social databases was conducted to find papers published between 1998 and 2009 (March). Given the broad nature of our review questions, we considered that this ten year period would generate considerable data that was within our resource capacity to analyse and that earlier papers would be covered in other systematic reviews. Databases were chosen for their coverage of mental health, primary health, psychosocial, health service and consumer content and included MEDLINE, Embase, Psychinfo, Cinahl, ProQuest, Sociological Abstracts, Family and Society Plus, Meditext and all Evidence Based Medicine (EBM) Reviews (which cover the Cochrane library databases and other evidence based medicine review databases). A range of search terms were used and adapted for each database based on an initial Medline search strategy (see additional file 1) and whether the database supported Medical Subject Headings (MeSH) or used other indexing terms.

The following operational definitions for primary mental health care and primary mental health care linkages formed the basis of the inclusion/exclusion criteria.

Primary Mental Health Care (PMHC) is:

1. Multi-faceted and comprising first level of contact, providing continuous care in a non-specialist setting. PMHC may include rehabilitation and ongoing support.

2. PMHC includes early intervention, treatment, health education and promotion for individuals as well as pathways to specialist care.

3. PMHC may include linkages with and referral between services in health (such as between a GP and mental health specialist) and non-health (such as with a welfare service).

4. PMHC concerns clinical care to individuals involving a primary health care clinician. While PMHC can include population-wide health promotion, advocacy and community development, these were not included in this review.

A primary mental health care linkage was defined as follows:

1. The linkage is the process used to connect two or more services in the provision of clinical primary mental health care.

2. One part of the linkage must involve a primary health care practitioner such as a GP, community nurse or practice nurse. The other part of the linkage can be any health or human service entity including hospital or community based mental health specialists, private practitioners, or non-health agencies such as housing, education or welfare etc. Linkages must be two-way which excludes a single referral without feedback or continuing relationship.

**Figure 1:**
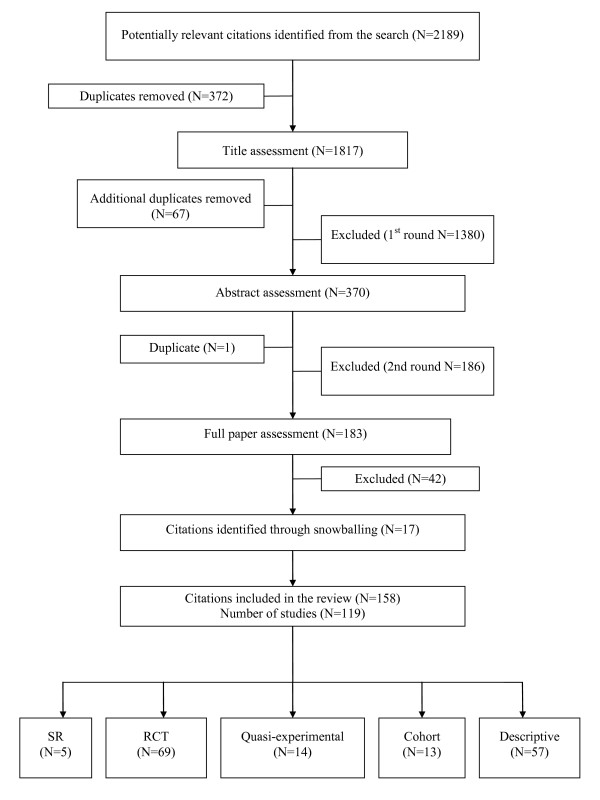
**Search results and process for selecting primary studies**.

Citations were included if the study provided evidence on ways that linked services demonstrated health gains or improved service provision; if the study was conducted in a comparable health system to Australia (UK, Europe, USA, Canada, New Zealand); if the article was available in English; and if the study was of analytic (randomised and controlled trials, cohort studies, case control studies, pre/post) or descriptive design (surveys, questionnaires, audits, case studies etc). All study types were included because we considered that description of linkage strategies and insights informing our second objective would likely be found in descriptive studies and qualitative papers. For this reason and because of the multiple study types included we did not exclude studies on quality and this limitation is discussed later. Commentaries, editorials, reviews of literature without systematic methodology, and opinion pieces, however, were excluded. Citations retrieved from the search (2189) were independently reviewed by two investigators (LH and SP) for inclusion first by title, and then by abstract and by full paper assessment as needed. Where there was disagreement or uncertainty, papers were assessed by a third investigator (JF, DP) and, if necessary, discussed by the team. Reference lists and included trials from systematic reviews were snowballed for relevance and citations assessed by two researchers (SP and JF). The final review data base comprised 158 papers covering 119 studies (see figure 1).

### Data extraction

Data extraction used a template designed for the project and was managed with an Access database. A coding framework was established to identify the linkage strategies reported in each study. Initial codes were set a *priori *based on the research team's knowledge and prior reading. The code list was refined throughout data extraction to make adjustment for the use of different terms for similar linkages and to add new linkages as required. Using this iterative process, the recurring linkages in the studies were identified and refined through team discussion. Studies were recoded where necessary to accommodate changes to the coding framework made during this iterative development. Studies were also coded according to the outcomes reported across clinical, service delivery or economic benefits. Research team members (JF, DP, LH and SP) independently coded and extracted data to the template from their share of allocated papers and a second team member checked and, if required, suggested an edit to the coding or data that was extracted.

### Outcomes assessed

We were interested in outcomes demonstrating clinical, service delivery and economic benefit. Indicators of clinical effectiveness included changes measured using validated instruments of mood, anxiety and other psychiatric symptoms; physical health outcomes; social functioning and quality of life. Physical health outcomes were included due to the numbers of people with mental health problems who have co-morbid chronic or debilitating physical symptoms.

Indicators of service delivery effectiveness included the following: treatment adequacy (access to and use of appropriate medications and duration of treatment); effective management (hospitalisation rates, bed days, referral rates to specialty services); treatment engagement (attendance at appointments, time to treatment etc); evidence based treatment (concordance with guidelines); quality improvement and evidence of improvement in organisational processes.

Economic outcomes were evaluated in two ways. First, where a significant positive clinical or service delivery outcome was reported at reduced or equivalent cost. Second, where a cost effectiveness analysis reported patient benefits (e.g. anxiety free days) at a cost similar to that of widely accepted treatments (such as standard treatments for elevated blood cholesterol levels).

### Analysis

Our analysis used a narrative and thematic synthesis approach because of the variability in linkages used and measurements reported. For the quantitative analysis of effectiveness reported in this paper, we focussed on the outcomes of the 42 randomised controlled trials (RCTs) of collaboration in primary mental health care. These studies provided the most rigorous evidence of effectiveness. Our synthesis of the data from these RCTs involved tabulating the proportions of trials where a significant result was reported.

## Results

### Link strategies identified in the peer reviewed literature

Ten linkages were found in the 119 studies and these are defined in table 1. As most studies used multi component linkage interventions, this resulted in a large number of combinations, which made it difficult to meaningfully interpret which combinations were the most effective. For this reason we thematically grouped strategies into four broad categories that comprised "direct collaborative activities", "agreed guidelines", "communication systems" and "service agreements". We then recoded the 42 randomised trials according to these broader linkage categories for comparison of linkages against outcomes.

**Table 1:** Classification of the linkages found in the review

Broad linkage category	Linkage	Definition
Direct collaborative activities	Link working	Organisational tasks connecting 2+ services - may involve limited clinical intervention but not expert clinical advice or structured liaison- does not include the work of an existing employed practice nurse undertaking extended tasks if there is no linkage work outside current general practice. Includes a process to clarify role between LW and others.
	
	Co-location	Face-to-face not virtual co-location- could lead to improved practitioner communication. Also includes MH worker (nurse, psychologist) located in primary care practice. Must be providing treatment, not simply an administrative arrangement.
	
	Consultation liaison	A practitioner connection where P1 has an explicit arrangement to provide expert level advice about ongoing care to P2 that is apart from the usual referral relationship - it may involve P1 receiving referral letters, making an assessment & providing some treatment and ongoing expert support to P2. Includes the specialists' advice to the primary care practitioner regarding treatment and monitoring (either directly or via another worker e.g., through link working) and may include educative roles. It does not involve the transfer of the patient from primary care.
	
	Care management	The coordination of care - it can include assessment, review and follow-up and a care management plan - linking with other services, or defined care pathway.

Agreed guidelines	Specific treatment protocols	An agreed process that is structured and documented about a specific patient treatment including evidence based algorithms such as in pharmacotherapy or Problem Solving Therapy in Primary Care (PST-PC). Does not include referral, stepped care or care management plan that are coded elsewhere.
	
	Stepped care	A treatment trajectory based on patient response or outcome. Involves a formal treatment escalation or de-escalation procedure to involve other providers based on specified patient outcomes.
	
Communication systems	Enhanced communication	A formal process with feedback -includes meetings, shared medical records, patient held records, consistent process for notifications, standardised letters, referrals and reports. May includes a worker from outside the practice attending the practice - e.g. to attend meetings.
	
	Enhanced referral	Expedited access, explicit referral criteria &/or process, which can include process for emergencies.
	
	Electronic communication system	Telephone or video communication between 2+ people with at least 2 practitioners not in same room - may or may not include patients. Includes 'telemedicine'.

Service agreement	Service or formal work agreement	Formalised contract or funding mechanism about how services will work together.

Note: the linkage strategies are activities and processes that are not mutually exclusive. For instance a psychologist may undertake link working activities and consultation liaison within their role while co-located in a primary care clinic.

### Indicators of clinical, service delivery and economic effectiveness

Usual care was the most common control. For the most part this involved some of the following components: the primary care physician received screening or diagnosis results; patients were notified of screening results; guideline specific treatment was promoted including annual screening with or without a treatment plan; other clinical information was provided; or patients could self-refer to the mental health services.

Depression trials provided the major evidence of clinical effectiveness (12 trials of depression and/or dysthymia and 2 trials of depression with an associated risk of drinking) [12-25]. Of these, nine trials concerned a general adult population and four a population aged 60 or over. The remaining 9 RCTs examined bipolar disorder [26], panic disorder [27,28], non specified 'serious or long term mental illness' [29-31] and mixed disorders [32,33].

As with clinical effectiveness, depression trials formed the largest number of RCTs showing a significant positive service delivery effect (10 trials of depression or dysthymia and one with associated alcohol risk) [18,34-37]. Of these, nine trials recruited a general adult population and two a population aged 60 or more. The remaining RCTs examined bipolar disorder [23], non-specified 'serious mental illness' [29], and first episode psychosis [38].

The economic data were limited. Studies used different measures for costs and benefits such as provider costs with no measure of benefits, through to studies that measured the costs per anxiety or depression-free day. Some studies apportioned intervention development costs by patient while others by primary care practice. Some studies reported lower overall costs associated with collaborative models [39,40], some reported no cost difference but improved clinical outcomes [13,28], and others reported improved clinical outcomes at higher costs which were comparable to the costs of treatments for other illnesses [41-43].

### Linkage strategies in studies with positive outcomes

The most common linkages in studies with a positive effect were care management, enhanced communication, consultation liaison and local protocols (see table 2). Analysis by the smaller number of broad linkage categories revealed that the most common combination with positive outcomes was "direct collaborative activities" plus "agreed guidelines" plus "communication systems". Studies using this combination reported a high proportion of positive clinical (81%) service delivery (78%) and economic (75%) outcomes (see table 3). Only the six combinations reported in the studies are shown in table 3. A lower proportion of studies that used linkages from a single broad category showed positive outcomes, compared to those studies that used linkages from multiple broad categories. Of all the broad categories, a "service agreement" was the only one not associated with any positive outcome.

**Table 2:** Number of randomised studies by linkage by reported significant outcome

Linkage	Clinical (number)	Service delivery (number)	Economic (number)
link working	8	5	3

co-location	6	4	3

consultation liaison	15	10	5

care management	18	11	8

specific treatment protocol	15	7	6

stepped care	3	2	1

enhanced communication	17	12	5

enhanced referral	3	5	0

electronic communication system	1	1	0

service or formal work agreement	0	0	0

Almost all linkages occurred as part of a combination strategy

**Table 3:** Number of randomised studies by broad linkage category by reported significant outcome

Broad linkage category	Number of studies	Clinical	Service delivery	Economic
Direct collaborative activities only	6	2/5	1/5	1/3

Direct collaborative activities + Agreed guidelines	5	3/5	1/2	2/3

Direct collaborative activities + Communication systems	10	5/9	5/5	3/5

Direct collaborative activities + Agreed guidelines + Communication systems	16	13/16	7/9	3/4

Direct collaborative activities + Communication systems + Service agreement	1	0	0	0

Communication systems only	4	0/2	1/4	0/1

**Total**	**42**	**23/37**	**15/25**	**9/16**

Number reporting a statistically significant positive outcome/the number assessing that outcome.

A descriptive account of linkages and outcomes can be gleaned from a closer examination of the two largest studies in the review, IMPACT and PRISM-E, as both of these are well described across a range of published papers. Both were conducted in the USA and used linkages from the "direct collaborative activities" plus "agreed guidelines" plus "communication system" suite. IMPACT employed Depression Care Specialists (DCS) who were nurses or psychologists with special study-related training who received expert supervision from a psychiatrist and primary care clinician [44-52]. The DCS role included working with patients and the primary care provider to conduct assessment, patient education, care management, Problem Solving Treatment in Primary Care and a relapse prevention plan. The treatment followed a stepped care process that was discussed at the team meetings. PRISM-E involved two forms of collaboration: integrated care clinics and off-site enhanced specialty referral services [53-59]. The integrated clinics co-located mental health and substance abuse specialists in primary care, whereas the enhanced speciality referral services involved referral to separately located mental health and substance abuse specialist services within two to four weeks.

On clinical effectiveness, patients receiving the IMPACT intervention fared significantly better than controls at every time-point and on every clinical outcome, except overall functional impairment at 24 months. The greatest differences were at 12 months. PRISM-E had a shorter follow up (six months) than IMPACT (24 months). Depression severity declined in both PRISM-E models but with a greater reduction in enhanced specialty referral, mainly due to the statistically significant reduction in depression severity for the sub-group with major depression.

On service delivery effectiveness a higher proportion of IMPACT patients compared to controls reported the use of any antidepressant medication or other treatment at every time point, but peaking at 12 months. Significantly higher use of depression treatment in the IMPACT patients at 18 and 24 months was accounted for entirely by pharmacotherapy. Differences in the use of counselling or specialty mental health care during the intervention ceased at 12 months. IMPACT patients reported greater confidence than controls in managing their depression (self efficacy) at 24 months. In PRISM-E higher treatment engagement was found in the integrated clinic as indicated by attendance for two or more visits, total number of visits and time to the first mental health visit.

On economic effectiveness the IMPACT study reported that the average cost per patient of the intervention was US$591, the incremental outpatient cost per depression-free day US$2.76, and the cost per QALY was $2519, which was thought similar to other mainstream treatments. The PRISM-E study did not report on economic effectiveness.

In addition to these two large RCTs, three of the five systematic reviews used meta-analyses and so provided convincing effectiveness data on collaborative care as well as some insight about the impact of particular linkage strategies. The cumulative meta-analysis of collaborative care by Gilbody et al [60] of 37 randomised trials of depression found that outcomes improved at six months, with evidence of longer-term benefit for up to five years. The main determinants of effect size were medication concordance and the professional background and method of supervision of case managers. Regular and planned supervision of the case manager by a specialist mental health clinician was related to a more positive clinical outcome. For most studies the supervision was provided by a psychiatrist, although it was not clear if it was the supervisor's expertise or discipline that was important. Gilbody et al concluded that sufficient evidence had emerged by 2000 to demonstrate a statistically significant clinical benefit from collaborative care, although this effect disappeared when the large trials from the USA were excluded from the analysis.

The meta analysis of 42 studies by Harkness and Bower [61] found that a positive service delivery outcome occurred when onsite mental health workers provided psychological and psychosocial interventions in primary care practices. On site mental health workers were associated with significant reductions in primary care provider consultations, psychotropic prescribing, prescribing costs and rates of mental health referral. Bower and Rowland [62] conducted a meta analysis of six trials that compared the clinical effectiveness of accredited counsellors located in primary care with usual care. They found greater clinical effectiveness in the counselling group in the short-term (one-six months) based on psychological symptom scores; however there was no difference at 12 months. There was also no difference between patients receiving counselling and those receiving usual care in terms of overall social function at any time point.

Butler et al [63] used forest plots of 25 quasi randomised and randomised controlled trials of depression to conclude that while there was evidence that integrated care improves some outcomes for persons with depression, the results were not consistent. They found that the majority of the studies showed significant benefit with regard to treatment response and remission, but only one model (IMPACT) showed consistent benefits in terms of symptom severity.

A narrative review of 38 trials of collaborative care by Craven et al [64] concluded that collaboration with treatment guidelines and systematic follow up was beneficial for people with depressive disorders. No direct relationship was found, however, between the degree of collaboration or efforts to improve medication adherence and clinical outcomes, but that enhanced patient education about their disorder generally showed good outcomes.

### Sub-group analysis

Patients with chronic and complex psychotic illnesses would be expected to have a high need for linked services and so we conducted a sub-group analysis. Sixteen of the 119 reviewed studies examined services for patients with psychosis including first presentations. Half used linkage strategies from the "direct collaborative activities" plus "communication systems" suite. These included use of link workers or other ways of providing comprehensive care management to patients, such as referral mechanisms for psychiatric support, liaison with associated health or welfare organisations, and monitoring and follow up using shared-care registers and patient-held records.

Nine studies examined clinical outcomes but only four used an RCT design. Three of these four reported some clinical benefit, such as improved mental and physical function with the use of a case manager [31], improved physical function with an integrated clinic [30] and reduced relapse with a quality program to improve team communication [29]. A comprehensive UK RCT by Byng et al (Mental Health Link) used facilitated meetings between general practice and community mental health workers, a link worker, registers, databases, audit and recall systems and payments to GPs [29]. The study reported that intervention patients had fewer psychiatric relapses and improved review and recall and intervention providers reported improved satisfaction. An RCT by Lester et al of a patient held record found no clinical or service use benefit [65].

There was some evidence in other studies of improved communication within co-located services and increased referral to mental health services [66,67]. However, a cohort study of a GP-community health team shared care register for patients with psychosis [68] showed no improvement in clinical outcomes or service use.

## Discussion

Most of the evidence supporting linkages in primary mental health care was generated from trials of adults with high prevalence disorders (usually depression). These trials reported clinical benefits such as symptom reduction, reduced severity, better treatment response, and improvements in physical and social functioning. Also reported were improvements in service delivery such as targeted referrals, reduced rates of hospitalisation and patient engagement with treatment, such as increased use of and self-efficacy with appropriate medication and adherence to other treatments. There was less evidence about service links for the low prevalence severe mental disorders (e.g. schizophrenia). We found very little evidence in the peer reviewed literature about primary mental health service links outside of the health sector (housing, employment and welfare) which would be most important for the implementation of a recovery model. The recovery model is a treatment concept where a service environment is designed so that patients have primary control over decisions about their own care [69]. While there are evaluations of such linkages in program reports, these have not yet been published in the peer-reviewed literature.

Our review provides strong support for the use of linkage combinations in primary mental health care. We developed a model of four broad linkage categories incorporating ten types of links that have been tested in the literature. The broad linkage categories were "direct collaborative activities", "agreed guidelines", "communication systems" and "service agreements".

The strongest body of evidence was for those interventions that used a combination of broad linkage categories that included at least one component from each of the "direct collaborative activities", "agreed guidelines" and "communication systems" suite. These were associated with statistically significant positive clinical, service delivery and economic outcomes. There was no evidence to support service agreements as either a single strategy or in combination with other strategies. These findings suggest that successful collaborative clinical programs in primary mental health care use multiple linkages that impact on the direct work of clinicians, more so than on management level agreement across services. Where studies assessed service delivery outcomes, the benefits over the long term were often attributed to medication concordance and a case manager with a health professional background and who received expert supervision.

Data on economic benefits were less conclusive due to differences in timeframes and economic indicators. However, three of the four studies that used linkages across the most common combination broad linkage category reported positive economic outcomes. Overall, just over a half of the economic studies reported that costs were lower, the same or acceptably higher given the additional clinical and service delivery benefits obtained.

The "successful" studies were sophisticated and complex, given the number of linkages to be developed and implemented simultaneously. Usual care was poorly described in many studies and as this was not standardised, it is impossible to know the effect of the therapeutic encounter between the patient and GP in the control arm of studies. Furthermore, if usual care itself does not conform with evidence based clinical guidelines and is "substandard" then it would not be difficult to demonstrate improvements above this usual care.

Our review adds new findings to the previous systematic reviews, in providing definitional description of the type of linkage strategies that have been trialed in primary mental health care. We have also examined which of these strategies were used in studies where effective outcomes were found. The cumulative meta-analysis reported by Gilbody et al, and Bower et al [60,70] demonstrated conclusively that collaborative care leads to better clinical outcomes, and that the important collaborative components were systematic identification of patients, professional background of staff and specialist supervision. The meta-analyses by Bower and Rowland [62] and Harkness and Bower [61] focussed on specific collaborative strategies, but in so doing, were not able to answer our research question about which strategies overall were associated with positive outcomes. Further research into the effectiveness of particular linkage strategies is warranted, such as to describe how strategies do operate in combination.

Most studies assessed outcomes up to six or 12 months and so the sustainability of programs and outcomes beyond this time is still largely unknown. The major trials included in our review and in previous systematic reviews were large, multi-centred USA studies. The Gilbody et al [60] review noted the influence of these trials, where the effect of collaborative care disappeared when USA studies were excluded from the meta-analysis. It cannot be assumed that what works in one country will work in another and a large trial is currently being conducted in the UK [71].

Almost all of the evidence in our review comes from separately funded studies in which additional research expertise is provided that is not usually available to services. Hence, rigorous evaluation of community programs is now needed to determine how successful initiatives can be implemented and sustained beyond short-term programs that are funded with additional research resources. Attention should now be paid to reviewing the evidence from these service evaluations that exist in reports outside the peer reviewed literature. Given the importance of the non health sectors, further research could also review the "grey literature" about what is known of links with other services, such as accommodation support programs [72]. Also our review did not cover primary mental health care in institutional settings, such as prisons, and so a further review would be warranted given the priority mental health needs of this group.

Our review has some limitations. The focus on developed nations with comparable health systems means that the findings may not be relevant to different and less well resourced national health systems. The search period of 1998-2009 was an arbitrary but reasonable timeframe given the trends in Australian health policy towards more integrated models over the preceding 10 years, and a desire to include all study types. Furthermore, we did not exclude based on study type, as this could have excluded valuable descriptive material about strategies and so we had a large amount of literature to review from this period. While it would have been useful to assess studies based on quality and weight the evidence across the range of studies, in order to obtain more rigorous evidence about which strategies are the most critical, this was made difficult by the incomplete and inconsistent manner in which linkages were described. Although we did limit the analysis of effective linkages to the 42 randomised trials, we did not apply further quality exclusion as our primary focus was on the linkage strategies, which were often not fully or consistently described and were differently implemented. Our method used to synthesise the data, by tabulating the proportions of trials where a significant result was reported, does not take into account differences in study power or the effect size. This means that we have attributed equal weight to the findings from the 42 trials. Within the broad purpose of the review, these limitations of study exclusion and analysis are acknowledged.

## Competing interests

The authors declare that they have no competing interests.

## Authors' contributions

JF & DP coordinated the review, conducted the data extraction and analysis and participated in the drafting of this paper. SP & LH conducted the search, data extraction, analysis and participated in the drafting of this paper. BK, RR, LM & LF advised on the conduct of the review and analysis and participated in the drafting of this paper. All authors read and approved the final manuscript.

## Pre-publication history

The pre-publication history for this paper can be accessed here:

http://www.biomedcentral.com/1472-6963/11/72/prepub

## Supplementary Material

Additional file 1 **Medline search strategy used for search and adapted for other databases**. List of search terms and sequence of search entries used in MEDLINE that was then adapted for use in other databases. Click here for file
