# Supplementary material for: Effectiveness of service linkages in primary mental health care: a narrative review part 1
Source: BMC Health Serv Res. 2011 Apr 11;11:72. doi: 10.1186/1472-6963-11-72 (PMC3079614; doi:10.1186/1472-6963-11-72)
Supplement: Additional file 1 — Medline search strategy used for search and adapted for other databases. List of search terms and sequence of search entries used in MEDLINE that was then adapted for use in other databases. [file 1472-6963-11-72-S1.DOC]

**Additional file 1: Medline search strategy used for search and adapted for other databases**

**Database: Ovid MEDLINE**Search Strategy:
--------------------------------------------------------------------------------
1     exp Primary Health Care/
2     exp Family Practice/
3     (primary adj (care or medic$ or practi$)).tw.
4     (general adj (practi$ or physician$)).tw.
5     or/1-4
6     exp Mental Health/
7     primary mental health.mp.
8     primary mental health care.mp.
9     *mental health services/ or *community mental health services/ or *social work, psychiatric/
10     (primary mental health adj (worker$ or nurse$ or clinic$ or practi$)).tw.
11     (primary mental health adj (service$ or counsel$ or therap$)).tw.
12     (community based adj (mental health or clinic$ or cent$)).tw.
13     or/6-12
14     5 and 13
15     exp "Referral and Consultation"/
16     *cooperative behavior/
17     exp "Delivery of Health Care, Integrated"/
18     *Partnership Practice/
19     *"Attitude of Health Personnel"/
20     exp Consumer Satisfaction/
21     *"outcome assessment (health care)"/ or *"process assessment (health care)"/
22     (mental health service$ adj (model$ or program$)).tw.
23     (service adj (link$ or integrat$ or partner$ or co-location$)).tw.
24     (collaborat$ adj (link$ or system$ or model$ or practi$ or clinic$ or care$)).tw.
25     (integrat$ adj (care or system or mental or health or service$ or model$)).tw.
26     (intersectoral adj (network$ or collab$ or link$ or mental health)).tw.
27     shared care.mp.
28     good shepherd model.mp.
29     or/15-28
30     14 and 29
31     limit 30 to (english language and humans and yr="1998 - 2009")
